# Norovirus Transmission Dynamics in a Pediatric Hospital Using Full Genome Sequences

**DOI:** 10.1093/cid/ciy438

**Published:** 2018-05-25

**Authors:** Julianne R Brown, Sunando Roy, Divya Shah, Charlotte A Williams, Rachel Williams, Helen Dunn, John Hartley, Kathryn Harris, Judy Breuer

**Affiliations:** 1 Microbiology, Virology and Infection Prevention and Control, Great Ormond Street Hospital National Health Service Foundation Trust; 2 Infection and Immunity, University College London, United Kingdom

**Keywords:** norovirus, epidemiology, molecular epidemiology, sequencing, whole genome

## Abstract

**Background:**

Norovirus is a leading cause of worldwide and nosocomial gastroenteritis. The study aim was to assess the utility of molecular epidemiology using full genome sequences compared to routine infection prevention and control (IPC) investigations.

**Methods:**

Norovirus genomes were generated from new episodes of norovirus at a pediatric tertiary referral hospital over a 19-month period (n = 182). Phylogeny identified clusters of related sequences that were verified using epidemiological and clinical data.

**Results:**

Twenty-four clusters of related norovirus sequences (“sequence clusters”) were observed, including 8 previously identified by IPC investigations (“IPC outbreaks”). Seventeen sequence clusters (involving 77/182 patients) were corroborated by epidemiological data (“epidemiologically supported clusters”), suggesting transmission between patients. Linked infections were identified among 44 patients who were missed by IPC investigations. Thirty-three percent of norovirus sequences were linked, suggesting nosocomial transmission; 24% of patients had nosocomial infections from an unknown source; and 43% were norovirus positive on admission.

**Conclusions:**

We show there are frequent introductions of multiple norovirus strains with extensive onward nosocomial transmission of norovirus in a pediatric hospital with a high proportion of immunosuppressed patients nursed in isolation. Phylogenetic analysis using full genome sequences is more sensitive than classic IPC investigations for identifying linked cases and should be considered when investigating norovirus nosocomial transmission. Sampling of staff, visitors, and the environment may be required for complete understanding of infection sources and transmission routes in patients with nosocomial infections not linked to other patients and among patients with phylogenetically linked cases but no evidence of direct contact.

## Teaser

Norovirus genome sequencing identified 33% of patients whose sequences were linked phylogenetically to those of another patient in the study. An additional 24% of nosocomially infected patients had unlinked sequences, suggesting infection from unsampled sources. Genome sequencing identifies unsuspected nosocomial norovirus infection.

Norovirus is a leading cause of gastroenteritis worldwide, associated with large outbreaks in healthcare facilities and with substantial clinical and economic implications [1–4]. The global financial burden is estimated at $60.3 billion annually [2]. Norovirus infections are typically self-limiting, with vomiting and diarrhea lasting 1–2 days. However, in immunocompromised patients, chronic infections can develop and last for weeks to years with considerable associated morbidity such as severe weight loss and malnutrition [5, 6].

Norovirus infections are primarily caused by genogroups GI and GII, each categorized into 9 and 22 genotypes, respectively (GI.1–GI.9 and GII.1–GII.22). Due to recombination between genotypes at the ORF1/ORF2 junction, norovirus has a dual typing system based on the polymerase (ORF1) and capsid (ORF2) sequences [7].

Partial genome sequencing of the hypervariable region of the capsid (P2) has previously identified nosocomial norovirus outbreaks in immunocompetent patients [8]. P2 sequences in transmission events are often identical [8, 9], with a 10% probability of 1–2 nucleotide changes in samples collected 3 weeks post-infection [10]. In immunocompromised patients, however, transmission may occur after in vivo evolution; several single-nucleotide polymorphisms (SNPs) are observed in the capsid sequence between linked patients [11]. When the infected population includes a high proportion of immunocompromised patients, as is the case at the tertiary referral children’s hospital in which our study was conducted, whole genome sequencing (WGS) may provide increased resolution to identify routes of transmission [12].

In this study we evaluated whether WGS can be used to better understand the sources of norovirus infection and transmission dynamics in a pediatric population with a high prevalence of immunocompromised patients.

## METHODS

### Study Population and Stool Samples

The study was carried out in a pediatric tertiary referral hospital with 350 beds, 60% of which are in single isolation rooms. There is no accident and emergency department; therefore, acute gastroenteritis is not the primary reason for admission unless it occurs in a patient already under the hospital’s care.

Stools from all symptomatic (diarrhea and/or vomiting) children (inpatient or outpatient) are tested for gastrointestinal viruses using real-time polymerase chain reaction (PCR), the methods for which are described elsewhere [13]. Norovirus-positive PCR results are reported as either norovirus GI or GII. Enhanced surveillance, with screening on admission and weekly for inpatients, is performed for all children admitted for hematopoietic stem cell transplant or congenital immunodeficiencies (symptomatic or asymptomatic). Details of the usual management of patients who are symptomatic on admission and any patients found to be norovirus positive by PCR are given in Supplementary Figure 1 and Supplementary Methods.

Norovirus infections detected less than 48 hours after admission to hospital are considered positive on admission (POA); those detected more than 2 days after admission are considered to have a nosocomial infection. Since the study hospital is a tertiary referral hospital, many patients have previously been admitted to local hospitals or had several outpatient visits prior to admission; earlier acquisition of infection in this or another healthcare facility cannot be excluded.

A nosocomial outbreak (“IPC [infection prevention and control] outbreak”) is suspected when 2 or more cases of gastroenteritis or confirmed positive norovirus cases occur within 48–72 hours in patients, staff, or visitors on the same ward or when an nosocomial infection occurs on the same ward as a chronically infected patient (Supplementary Figure 1). An outbreak meeting is called and standardized IPC outbreak measures are implemented (Supplementary Figure 1 and Supplementary Methods). The index is presumed to be the person in whom norovirus was first detected, unless a point source is suspected.

In this study residual specimen from the first positive sample from all norovirus-positive patients between 1 July 2014 and 17 February 2016 (19 months) was submitted for WGS. A total of 205 norovirus PCR-positive patients were identified during the study period. Ten had no residual specimens and 6 were PCR negative on re-extraction. The remaining 189 samples were whole genome sequenced. The median patient age was 2 years (range, 1 month to 16 years); 59% of patients were profoundly immunocompromised with primary immunodeficiency syndromes, solid or bone marrow transplants, or receiving chemotherapy for malignancies.

### Norovirus Whole Genome Sequencing

RNA purification, cDNA synthesis, and full genome sequencing using SureSelect target enrichment followed by de novo assembly were carried out as described previously [14]. A consensus sequence was generated. To verify correct genome assembly, open reading frames (ORF-1, ORF-2, and ORF-3) were identified for all sequences using the Find Open Reading Frames tool in CLC Genomics Workbench (v 9.0) [14].

### Genotyping and Phylogenetic Analysis

In total, 184/189 samples generated greater than 90% genome coverage and >100-fold read depth. Full genome consensus sequences were submitted to the Norovirus Genotyping Tool [15] to determine the genotype.

Two sequences were excluded from phylogenetic analysis as infection with a mixture of genotypes was detected and a robust consensus sequence could not be generated [14]. Phylogenies were reconstructed in CLC Genomics Workbench (v 9.0) from the remaining 182 consensus sequences, as described in Supplementary Methods.

### Data Mining to Establish Epidemiological Support of Sequence Clusters

Patients whose norovirus genomes branched together in a phylogenetic tree from a single common ancestral node (monophyletic), with absent or short branch lengths and a high probability that the branching order was correct (high bootstrap value), were referred to as “sequence clusters.” To determine whether these could be due to nosocomial transmission, inpatient and outpatient records together with norovirus PCR laboratory result histories for each patient were retrieved. Patients within a sequence cluster were considered to be epidemiologically linked if they were norovirus positive with an inpatient or outpatient visit that overlapped by at least 24 hours; these were referred to as “epidemiologically supported clusters.” Sequence clusters that were not supported by epidemiological evidence were referred to as “epidemiologically unsupported clusters.”

## RESULTS

### Norovirus Infections and Genotypes

During a 19-month period (1 July 2014–1 February 2016) we generated full genome sequences for 182 new norovirus episodes in a pediatric tertiary referral hospital with a high prevalence of immunocompromised patients. Of these, 84 were based on routine IPC investigations and considered to be nosocomial infections. During this period, 8 IPC outbreaks of norovirus were identified (Table 1), which accounted for only 37/84 of the nosocomial infections. To confirm whether the declared outbreaks were truly linked and to better understand the sources of infection for the remaining episodes, we reconstructed phylogenetic trees from all 182 sequenced norovirus genomes. Phylogenetic trees reconstructed from GII.4 partial capsid sequences (the hypervariable P2 region used in routine typing methods) provided insufficient information to identify 2 of the 10 sequence clusters obtained using full genomes and missed 14 of the 37 (38%) patients linked by full genome sequences (Supplementary Figure 2). Moreover, while phylogenetic analysis using full genome sequences is well supported, with 77% (55/71) of internal nodes in the whole genome tree supported by bootstrap values ≥70, maximum likelihood phylogeny using the hypervariable capsid P2 domain sequences (427 nt) generated a tree with low bootstrap support. Only 34% (24/71) of internal nodes were supported by bootstrap values ≥70 (Supplementary Figure 3). Separate phylogenies reconstructed for ORF1 and ORF2 sequences showed no difference in branching order. This suggests that recombination at the ORF1/ORF2 junction, the most frequently described breakpoint in norovirus recombination events [16, 17], did not occur within the sampled population. Therefore, all further analysis was performed using full genomes.

**Table 1.** Sequence Clusters Identified by Maximum Likelihood Phylogeny Using Full Genome Sequences

Sequence Cluster Number	Genotype	Number of Patients	Date Range	Number of Wards	Number of Clinical Specialties Involved	Bootstrap Support	Diversity Within Cluster^a^	Identified by Infection Prevention and Control Investigation	Supported by Classic Epidemiology^b^
4	GII.P7_GII.6	3	7 days	1	1	100	0	Yes	Yes
5	GII.P21_GII.3	17	3 months	6	3	100	0–22	Partially	Yes (16/17)
6	GII.P21_GII.3	2	3 days	1	1	82	14	No	Yes
7	GII.P21_GII.3	6	1 month	3	2	70	0–10	Partially	Yes
8	GII.P21_GII.3	2	2 months	1	1	100	11	No	Yes
9	GII.P21_GII.3	2	2 days	1	1	100	12	No	Yes
10	GII.P21_GII.3	9	17 months	2	1	100	19–149	Partially	Yes
23	GII.P21_GII.3	2	3 months	2	2	100	29	No	Yes
11	GII.Pe_GII.4	8^**c**^	2 months	2	2	100	1–24	Partially	Yes
12	GII.Pe_GII.4	2	6 days	1	1	100	3	No	Yes
13	GII.Pe_GII.4	2	3 days	1	1	100	0	No	Yes
14	GII.Pe_GII.4	4	11 days	2	1	100	1–4	Partially	Yes
15	GII.Pe_GII.4	3	3 days	1	1	100	1–3	Yes	Yes
16	GII.P4_GII.4	7	3 months	2	1	100	0–35	Partially	Yes
17	GII.P4_GII.4	2	25 days	2	1	100	14	No	Yes
18	GII.P4_GII.4	5	2.5 months	3	2	77	0–25	No	Yes
19	GII.P4_GII.4	2	19 days	1	1	100	6	No	Yes
1	GI.P3_GI.3	2	8 months	2	2	100	31	No	No
2	GII.P2_GII.2	2	2 months	2	2	100	7	No	No
3	GII.P7_GII.6	2	3 months	2	2	100	17	No	No
20	GII.Pe_GII.4	2	5.5 months	2	2	100	12	No	No
21	GII.P21_GII.3	3	4 months	3	1	95	17–28	No	No
22	GII.P21_GII.3	2	6 months	2	2	98	36	No	No
24	GII.P21_GII.3	2	3 months	2	1	100	18	No	No

All GII.4 capsid genotypes are Sydney 2012. GII.4 polymerase genotypes are GII.Pe Sydney_2012 or GII.P4 New Orleans_2009.

^a^Expressed as the number of nucleotide differences across the whole genome.

^b^Overlap in norovirus-positive period and hospital attendance.

^**c**^Including 1 parent (NORO/51, father of NORO/52).

**Figure 1.**
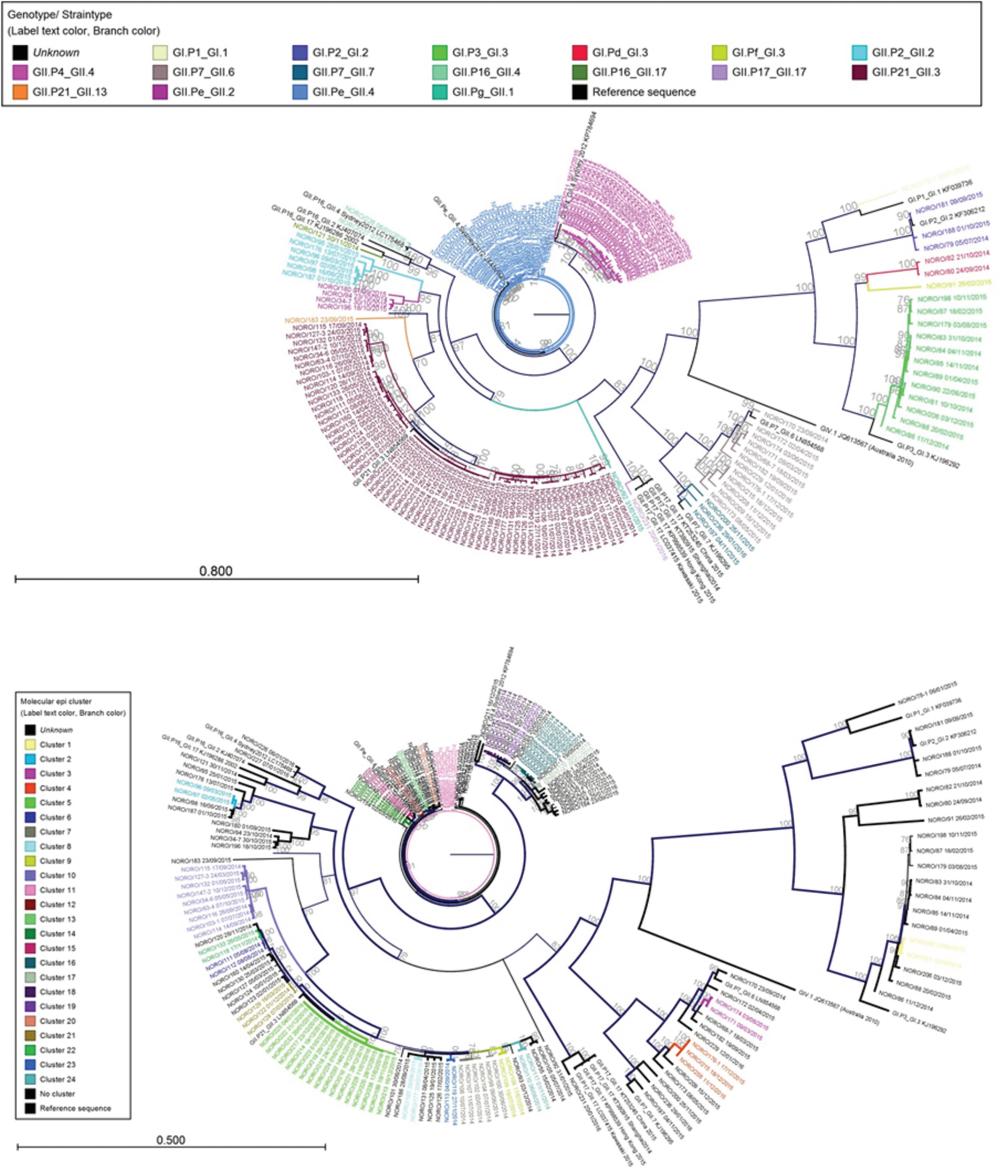
Maximum likelihood phylogeny of full genome sequences from norovirus episodes over a 19-month period (2014–2016) color coded by genotype *(a*) and color coded by sequence cluster number *(b*). Separate maximum likelihood phylogenies for each genotype with sequence cluster, number annotated and displaying greater resolution *(b*), are shown in Supplementary Figure 5.

A total of 11 capsid and 14 polymerase genotypes were identified in 17 unique combinations (Figure 1, Supplementary Figure 4).

### IPC Outbreaks and Sequence Clusters

All (8/8) previously identified IPC outbreaks corresponded to monophyletic sequence clusters identified by phylogenetic analysis (Supplementary Figure 5). An additional 23 patients for whom no source of infection had previously been identified were linked by phylogenetic analysis to 6 of the IPC outbreaks (Table 1). Eight of the 23 patients were norovirus POA but had had previous inpatient stays or outpatient appointments during which transmission could have occurred. Four patients previously identified as part of 3 of the outbreaks were shown to have sporadic infections with unrelated genotypes. All 4 patients had nosocomial infections of unknown origin.

Phylogenetic analysis identified 16 new sequence clusters involving 36 patients that had not previously been identified by IPC investigations, including 6 patients who were norovirus POA but had had previous inpatient stays or outpatient appointments during which transmission could have occurred. In total, 24 sequence clusters were identified by phylogenetic analysis comprising 2–17 sequences (median 2) per cluster (Table 1, Supplementary Figures 5 and 6, and Supplementary Methods).

### Epidemiological Support for Sequence Clusters

Review of inpatient and outpatient histories together with the timing of norovirus shedding identified plausible links between patients within 17 of the 24 sequence clusters (including the 8 IPC outbreaks originally identified by IPC investigations) (Supplementary Figures 6 and 7). These epidemiologically supported clusters confirm nosocomial transmission among 77/92 (84%) of the patients linked in sequence clusters, of whom only 33 had been previously identified by IPC methods (Table 2). The epidemiologically supported clusters were caused by genotypes GII.P21_GII.3 (7/17 clusters), GII.Pe_GII.4 (5/17), GII.P4_GII.4 (4/17), and by GII.P7_GII.6 (1/17). Of the 44 patients in epidemiologically supported clusters who were missed by IPC investigations, 11/44 were on the same ward but involved only 2 patients each, 7/44 were on a different ward with a shared clinical team, and 21/44 occurred over a prolonged period of time.

**Table 2.** Comparison of Norovirus Transmission Events Identified by Phylogenetic Analysis (Epidemiologically Supported Clusters) and Classic Infection Prevention and Control (IPC) Investigations (IPC Outbreak)

	Part of IPC Outbreak	Not Part of IPC Outbreak	Total
Transmission inferred by molecular epidemiology	33	*44 * ^ a^	77
No transmission inferred by molecular epidemiology	*3 * ^ b^	102	105
Total	36	146	182

Nonitalicized text indicates patients correctly assigned by IPC investigations. Italicized text indicates patients incorrectly assigned by IPC investigations.

Abbreviation: IPC, infection prevention and control.

^a^Including 1 patient who was incorrectly assigned to an outbreak by IPC investigations (cluster 11), but shown by molecular epidemiology to be a different genotype from the rest of the outbreak and linked to another patient in cluster 23.

^b^Shown to be a different genotype from the rest of their respective IPC outbreak.

For the remaining 7 (of 24) sequence clusters, none of which were identified by IPC investigations, no evidence of an epidemiological link could be found between patients in each cluster, including when the residential postcodes of patients were examined to assess whether community transmission could have occurred. These epidemiologically unsupported clusters were caused by GII.P21_GII.3 (3/7), GII.Pe_GII.4 (1/7), GI.P3_GI.3 (1/7), GII.P2_GII.2 (1/7), and GII.7_GII.6 (1/7).

### Sources of Infection

In total, 103/182 patients had nosocomial infections, of whom 84 were identified based on routine IPC investigations. No source could be identified for 43/84 patients (Table 3), including 9 whose infection occurred at the beginning of the study and who therefore may have been infected by a patient not included in the study. The remaining 19/103 patients with nosocomial infection were originally classified as norovirus POA. However, all were linked by phylogenetic analysis to other patients in epidemiologically supported clusters; they were all subsequently found to have attended the hospital in the recent past. Seventy-nine patients (79/182) were norovirus POA (Table 3) and were not linked to any other sequences in the phylogenetic analysis. This included 4 patients who were index cases in subsequent onward transmissions.

**Table 3.** Summary of Sources of Infection at Pediatric Tertiary Referral Hospital During Study Period, July 2014–February 2016

Source of Infection	Number of Patients, n = 182 (%)	Proportion of Patients Immunocompromised (%)
Another patient (part of an epidemiologically supported cluster)	60 (33)	45/60 (75)
Outside of the hospital (norovirus positive on admission)	79 (43)	37/79 (49)
Unknown (nosocomial infection but not transmission from another patient in the study^a^)	43 (24)	27/43 (63)

Epidemiologically supported clusters are sequence clusters identified by phylogenetic analysis and supported by epidemiological evidence.

^**a**^Not part of a monophyletic sequence cluster (with <38 single-nucleotide polymorphisms within cluster) and therefore not linked to any other virus sequences within the study cohort.

### Pairwise Distances Between Genome Sequences

**Figure 2.**
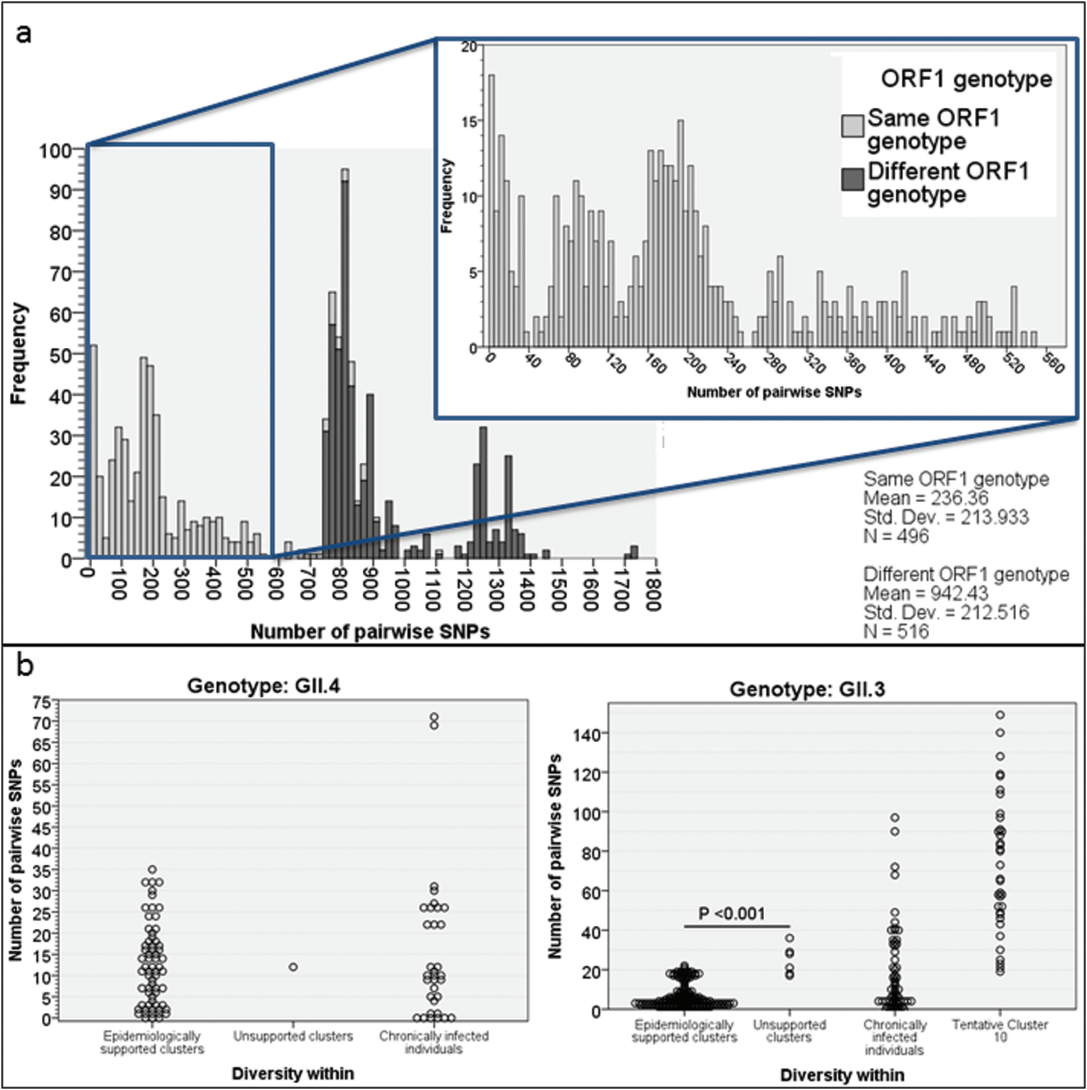
Pairwise distances in GII.3 and GII.4 epidemiologically supported clusters, epidemiologically unsupported clusters, and longitudinally sampled chronically infected patients. Epidemiologically unsupported clusters are those identified by phylogenetic analysis but not supported by classic epidemiological evidence. (*a*) Pairwise differences between local and database whole genome sequencing. (*b*) Pairwise differences plotted by the category of cluster (epidemiologically supported or unsupported and longitudinally sampled individuals) into which they fall. The greater pairwise differences within cluster 10 are shown. Abbreviation: SNP, single-nucleotide polymorphisms.

The pairwise distances (Supplementary Methods) between GII.4 norovirus genomes from each patient demonstrated a distinct population of sequences with ≤38 SNPs difference (Figure 2a), which corresponds to >99.5% sequence identity across the genome and falls within the range of previously described within-host SNP diversity seen in longitudinally sampled chronically infected patients [18] (Figure 2b). This equates to the cutoff of ≤2 SNPs (99.5% identity) where the partial capsid P2 domain has been used to identify transmission events among norovirus GII.4 infections in immunocompetent patients [10, 11]. Consequently, in this study, sequence clusters were identified if groups of full genome sequences formed a monophyletic cluster with fewer than 38 pairwise SNPs between them. As controls, we included pairwise distances between sequences collected from repeatedly sampled chronically infected individuals [18].

The mean pairwise distance for 62 GII.3 and 71 GII.4 sequences comprising the majority of the epidemiologically supported clusters was 7 SNPs for the former (range, 0–22) and 12 SNPs for the latter (range, 0–35). The mean pairwise distance in the GII.3 and GII.4 epidemiologically unsupported clusters was 25 SNPs for the former (range, 17–36) and 12 SNPs for the only GII.4 epidemiologically unsupported cluster (Figure 2b). Thus, pairwise differences were significantly higher for epidemiologically unsupported compared with epidemiological supported GII.3 clusters (*P* < .001, mean difference 18 SNPs, 95% confidence interval of the difference 13–23 SNPs; 2-tailed *T* test, SPSS v24).

The results were skewed by the epidemiologically supported cluster, GII.P21_GII.3 cluster 10 (Supplementary Figure 6). Sequence cluster 10 was monophyletic with strong epidemiological support but higher pairwise distances (19–149 SNPs). However, all patients were located on the same or on a linked ward, managed by the same clinical team and with overlapping admissions and norovirus shedding (Supplementary Figure 6c). The sequence cluster could not be disrupted by inclusion of all publically available GII.P21_GII.3 sequences in the phylogenetic analysis (Supplementary Figure 8), making it unlikely that this sequence cluster represents repeated introductions to the hospital from an external source. We have previously shown a linear relationship between the number of SNPs and duration of infection in chronically infected immunosuppressed patients [18]. The diversity within epidemiologically supported cluster 10 is therefore likely to have arisen due to virus evolution in this patient group, the majority (8/9) of whom were immunosuppressed and chronically infected for extended periods (38–388 days) between transmission events.

## DISCUSSION

Information on the routes of norovirus transmission in a nosocomial setting is necessary for allocation of IPC resources and effective containment of infection. While capsid P2 sequencing is currently the standard method for norovirus molecular epidemiology, we show that it is not sufficiently discriminating for robust investigation of putative transmissions among immunosuppressed patients. Here, we demonstrate that WGS is superior to both P2 and IPC methods for ascertaining transmission among immunosuppressed patients, having identified 44 patients not previously known from IPC investigations to be part of transmission chains.

For 60/103 nosocomial infections, phylogenetic analysis showed linkage to another patient in the study. However, for the remainder, including the index patients in 13/17 epidemiologically supported sequence clusters, the sources were unknown and potentially included unsampled staff, visitors, or patients. Within epidemiologically supported clusters, 75% of patients were profoundly immunocompromised and managed in single isolation rooms with limited or no direct contact between patients, making the route of transmission unclear. Additional sampling could help here and may also shed light on the 7/24 epidemiologically unsupported clusters, which accounted for 14 of the 93 (15%) patients identified as linked by phylogenetic analysis. Of interest for non-GII.4 norovirus genotypes that do not spread as pandemics, Parra et al [19] observed a “static” pattern of diversification, with only a few residue changes over several decades. Thus, for the unsupported non-GII.4 clusters, it is possible that due to a lack of genome variability, independent episodes of norovirus infection appear linked based on phylogenetic analysis alone. This needs additional investigation.

Also, we attempted to verify sequence clusters by calculating pairwise SNPs for sequences within a monophyletic sequence cluster. While epidemiologically supported GII.4 clusters all had <38 SNPs between sequences and most epidemiologically supported GII.3 clusters had <22 SNPs, the epidemiologically supported GII.3 cluster 10 fell outside this range, probably because of within-host evolution prior to transmission among chronically infected patients. This should be noted when using genomics to manage outbreaks (Supplementary Figure 9).

Norovirus is now the major cause of acute gastroenteritis worldwide [2], with morbidity and mortality seen in infants in low-income countries and the elderly and in immunosuppressed patients in middle- and high-income countries [6]. IPC is critical to managing disease, particularly in hospitals and other institutions. However, for immunosuppressed patients, in particular, the sensitivity of routine IPC investigations alone for identifying linked transmission is 44% compared with IPC plus WGS (Table 2). While 33% of new norovirus cases in this study were acquired from another patient, despite isolation nursing and stringent IPC measures, the source of infection for 43% of nosocomial infections remains unknown even with WGS, pointing to the need for wider sampling of patients, staff, visitors, and the environment. Nonetheless, WGS could be a valuable tool with which to focus IPC interventions in areas of the hospital where nosocomial acquired infections are most problematic. With ever decreasing sequencing costs and technologies that allow rapid turnaround times [14], the possibility that norovirus genome sequencing, perhaps linked to electronic patient records, could be used routinely to control nosocomial infections is now a reality.

## Supplementary Data

Supplementary materials are available at *Clinical Infectious Diseases* online. Consisting of data provided by the authors to benefit the reader, the posted materials are not copyedited and are the sole responsibility of the authors, so questions or comments should be addressed to the corresponding author.

## Supplementary Material

Supplementary Figures

Supplementary Methods

Supplementary Figure

## Notes

***Acknowledgments.*** We acknowledge the work and support of the Great Ormond Street Hospital for Children NHS Foundation Trust Infection Prevention and Control team. We acknowledge the infrastructure support from the University College London (UCL) Pathogen Genomics Unit, the National Institute for Health Research (NIHR) UCL/University College London Hospital (UCLH) Biomedical Research Centre, and the UCL Medical Research Council Centre in Molecular Medical Virology. All research at Great Ormond Street Hospital NHS Foundation Trust is made possible by the NIHR Great Ormond Street Hospital Biomedical Research Centre. The study was approved by the National Research Ethics Service Committee London–Brent (Research Ethics Committee reference 14/LO/1331).

***Financial support.*** This work was supported by the PATHSEEK European Union’s Seventh Programme for Research, Technological Development and Demonstration (grant 304875) and a National Institute for Health Research (NIHR) doctoral fellowship (grant NIHR-HCS-D12-03-15) to J. R. B. It is noted that J. B. receives funding from the NIHR UCL/UCLH Biomedical Research Centre. The views expressed are those of the author(s) and not necessarily those of the NHS, the NIHR, or the Department of Health.

***Potential conflicts of interest.*** All authors: No reported conflicts of interest. All authors have submitted the ICMJE Form for Disclosure of Potential Conflicts of Interest. Conflicts that the editors consider relevant to the content of the manuscript have been disclosed.
