# Supplementary material for: Norovirus Transmission Dynamics in a Pediatric Hospital Using Full Genome Sequences
Source: Clin Infect Dis. 2018 May 25;68(2):222–8. doi: 10.1093/cid/ciy438 (PMC6321856; doi:10.1093/cid/ciy438)
Supplement: Supplementary Figures [file ciy438_suppl_supplementary_figures.docx]

Norovirus PCR positive patient(s) and/or symptomatic staff or carers

Instigate control measures (see methods)

Same genogroup (GI / GII)?

Hospital acquired?

(symptomatic / PCR + >48 hrs after admission)

Same ward?

≥ 2 hospital acquired cases within 48-72 hr period

Suspected “IPC outbreak”

Outbreak control meeting and actions

Yes

Yes

No outbreak

No

No outbreak

No outbreak

No outbreak

Yes

No

Yes

No

Yes

Supplementary Figure 1 : Infection Prevention and Control Decision tree following detection of Norovirus infection

No

**Supplementary Figure 2.** Maximum likelihood phylogeny of (a) full genome and (b) hyper-variable capsid P2 domain (427 nt) sequences for norovirus GII.4 episodes during study. Cluster numbers refer to sequence clusters identified using full genome sequences. Sequences are labelled with a unique patient identifier and the specimen collection date. Fewer clusters (8/10) and fewer linked patients (23/37) are identified using P2 sequences compared to full genomes.
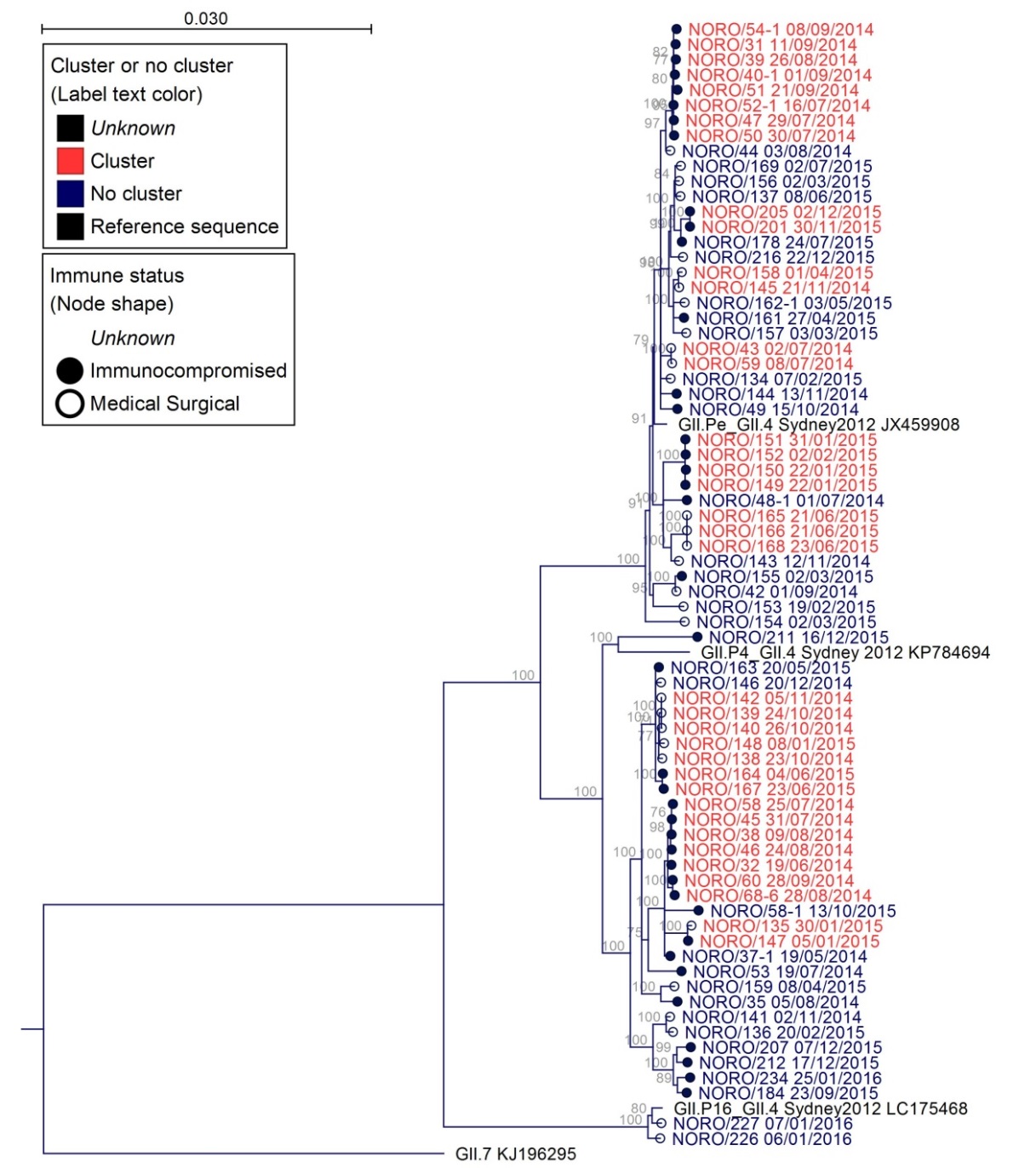

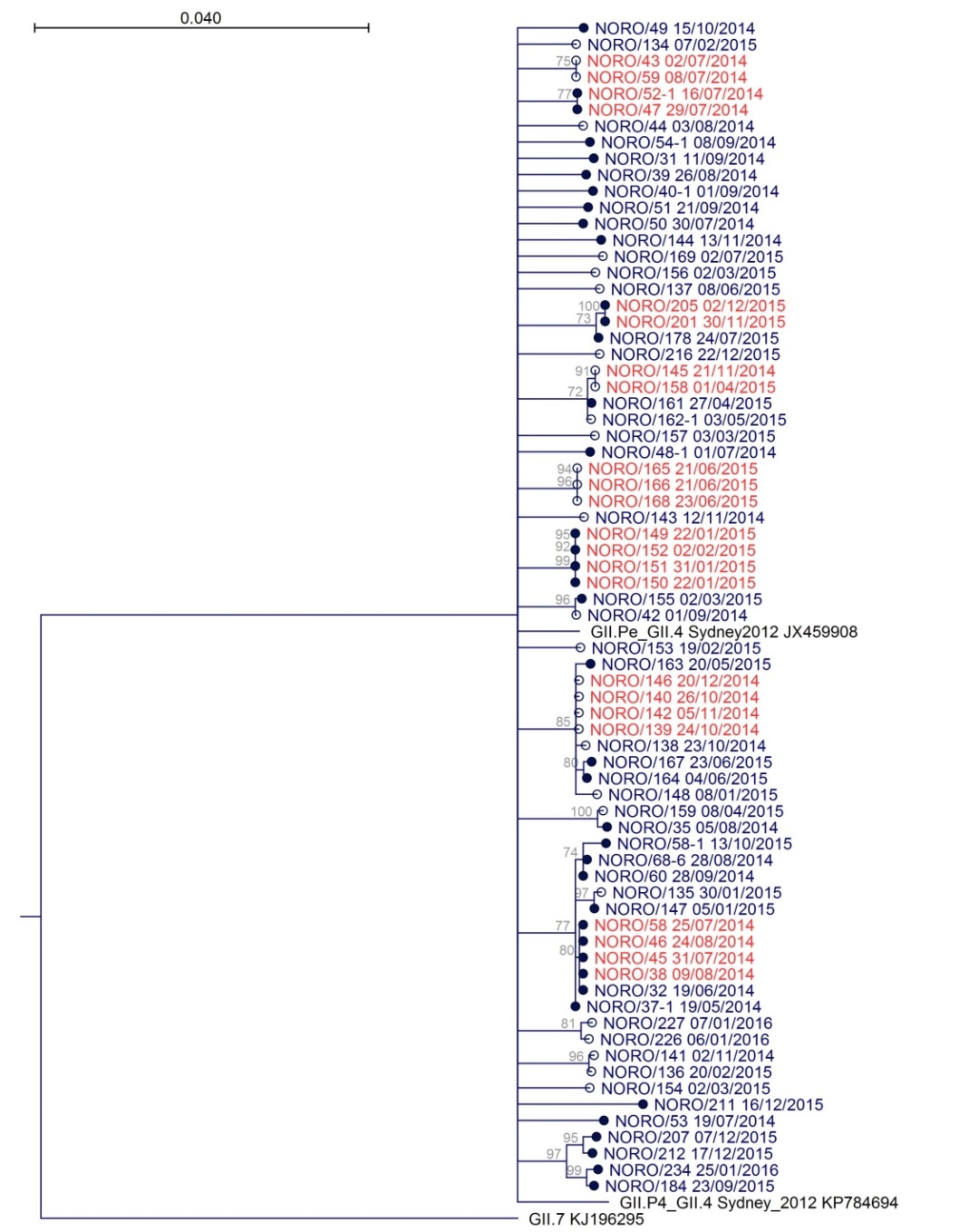


**Cluster 11**

**Cluster 12**

**Cluster 20**

**Cluster 14**

**Cluster 15**

**Cluster 18**

**Cluster 19**

**Cluster 16**

**Cluster 17**

**Cluster 16**

**Cluster 11**

**Cluster 12**

**Cluster 13**

**No cluster**

**Cluster 18**

**Cluster 14**

**Cluster 15**

**Cluster 20**

**Cluster 13**

**(b)**

**(a)**

**Supplementary Figure 3.** Frequency histogram of internal node bootstrap support values in norovirus GII.4 maximum likelihood trees using full genomes or P2 domain sequences. Phylogenetic analysis using full genome sequences is well supported, with 77% (55/71) of internal nodes in the whole genome tree supported by bootstrap values ≥70. Maximum likelihood phylogeny using the hyper-variable capsid P2 domain sequences (427 nt) generates a tree with low bootstrap support; only 34% (24/71) of internal nodes are supported by bootstrap values ≥70.

**Supplementary Figure 4.** Norovirus genotypes identified in paediatric tertiary referral hospital (GOSH), July 2014 – February 2016 (n = 184).


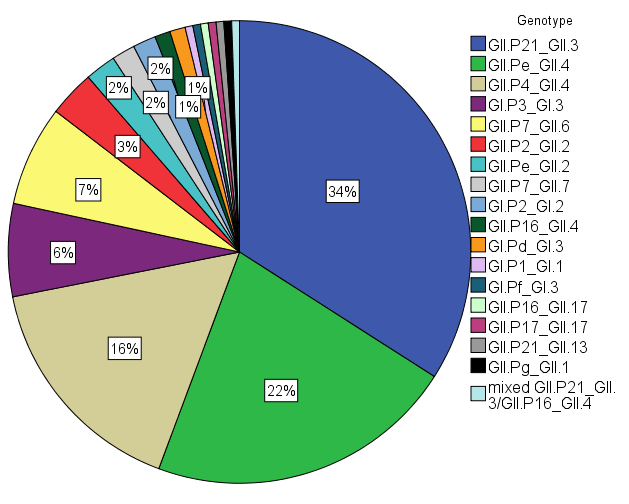


**Supplementary Figure 5.** Maximum likelihood phylogeny of full genome sequences from norovirus episodes with **(a)** GI.2 , **(b)** GI.3, **c)** GII.2, **d)** GII.6, **e)** GII.3, **f)** GII.17 and **(g)** GII.4 sequences. Individual sequences are labelled with a unique patient identifier (NORO/XX) and specimen collection date and colour coded according to the ward on which the patient was located. Cluster numbers correspond to sequence clusters in Table 1.

**(a)**


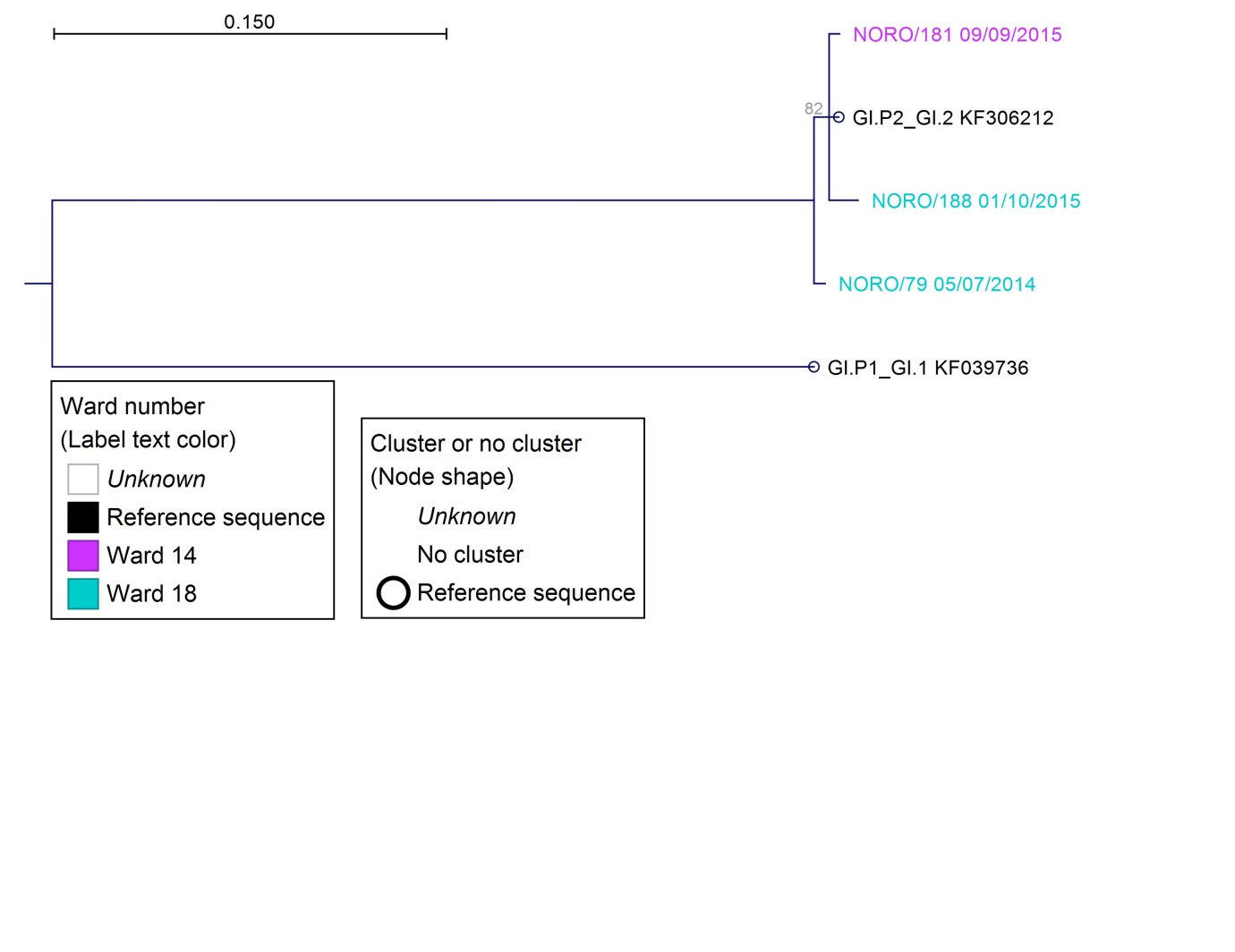


**(b)**


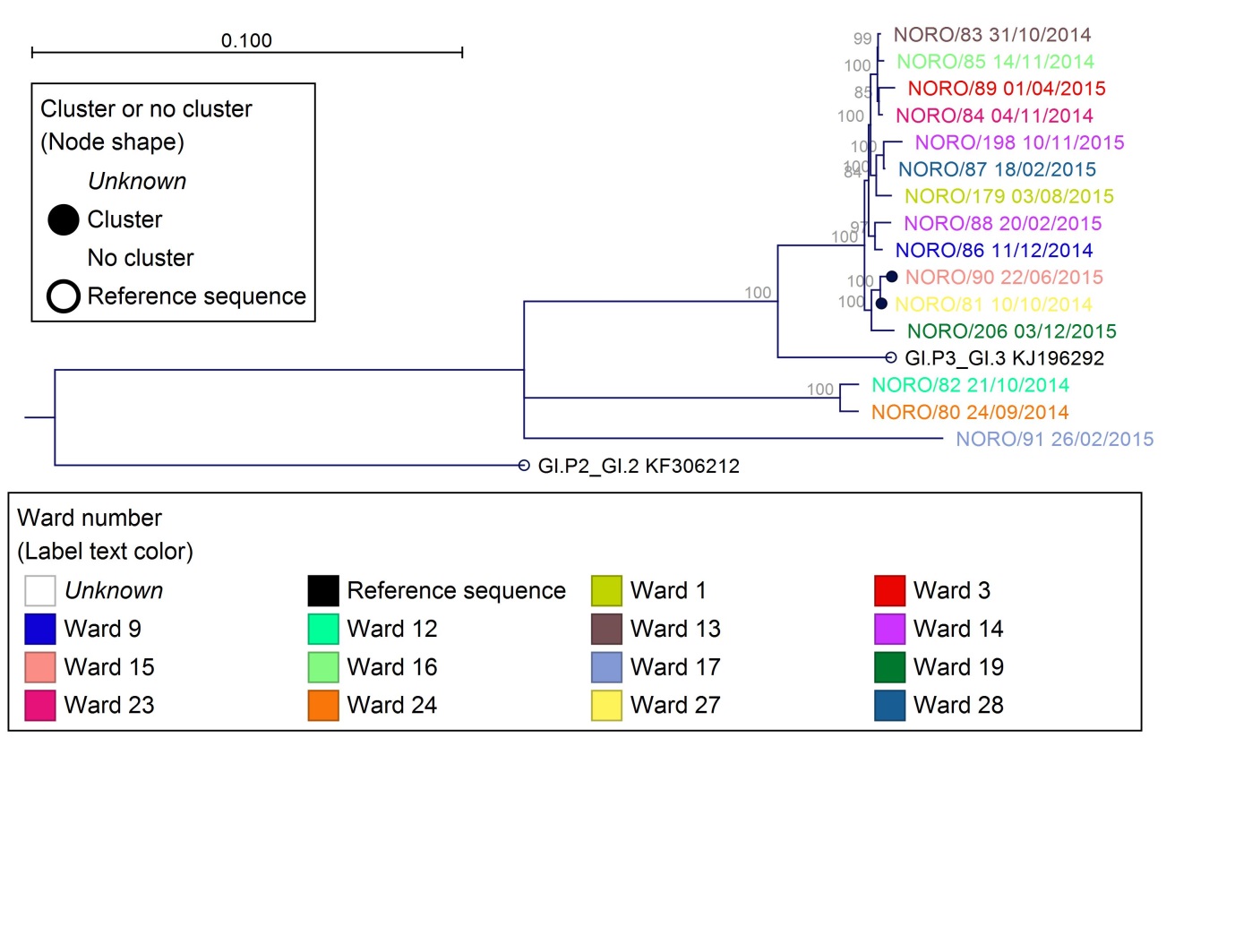


**Cluster 1**

**(c)**


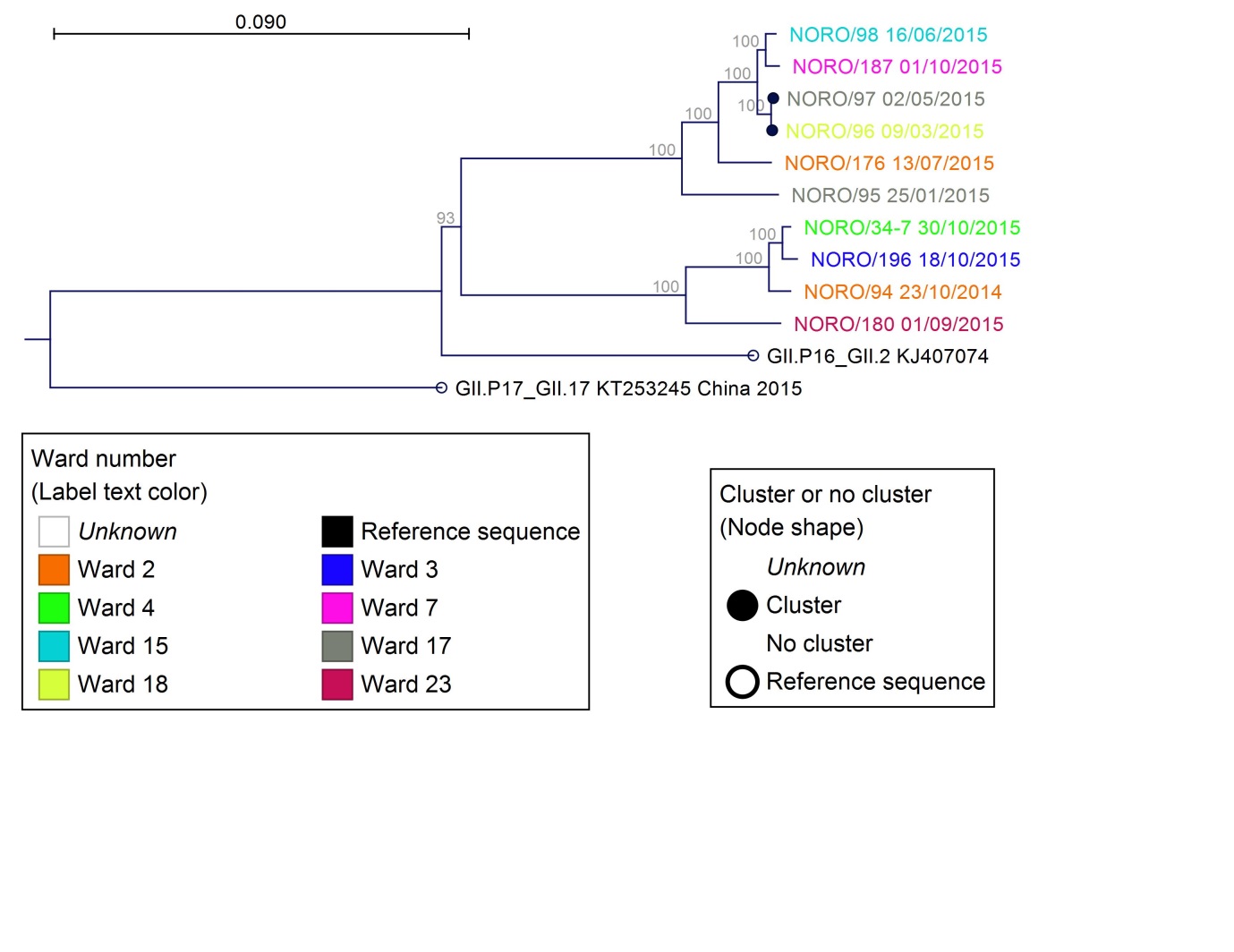


**Cluster 2**

**(d)**


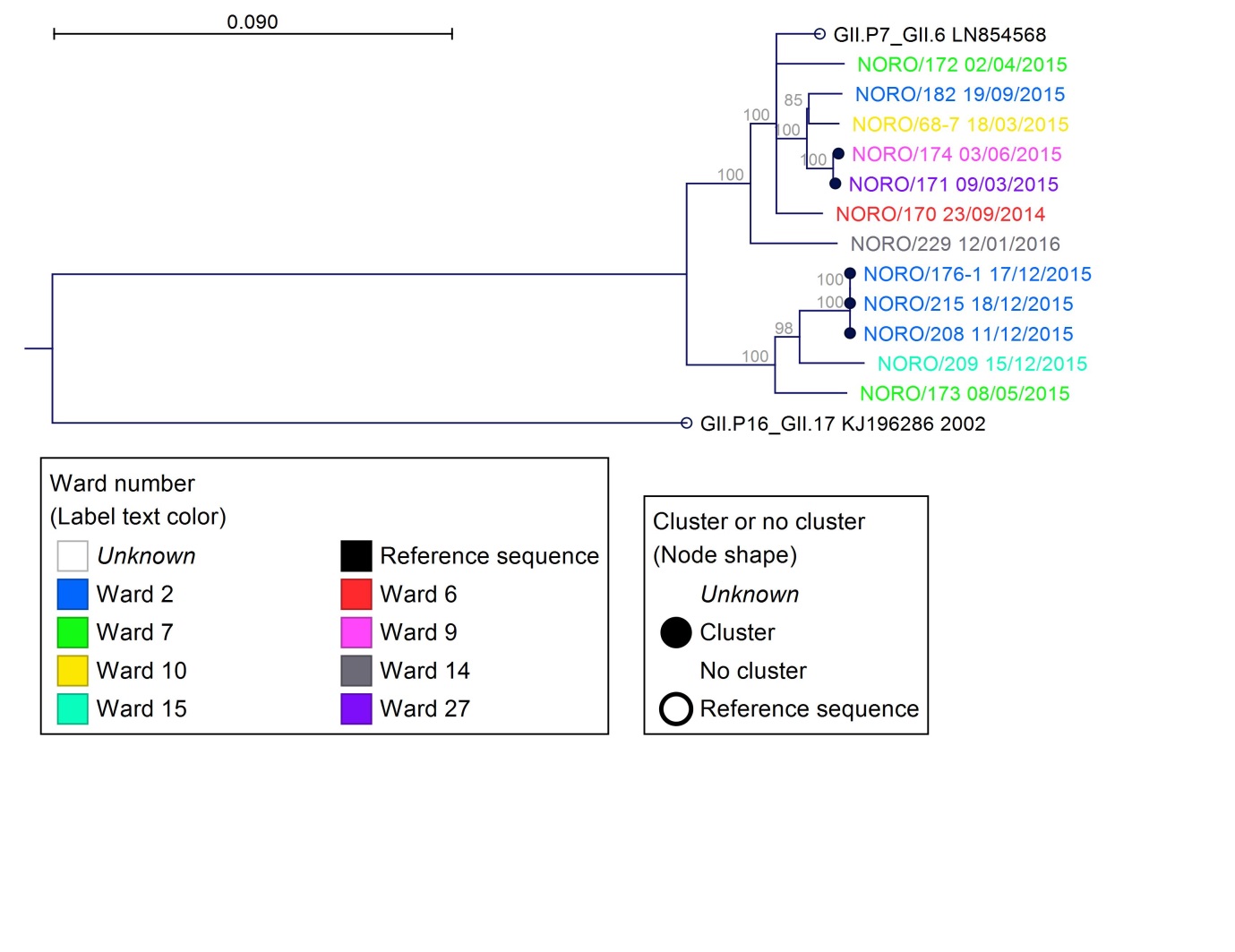


**Cluster 4**

**Cluster 3**

**(e)**


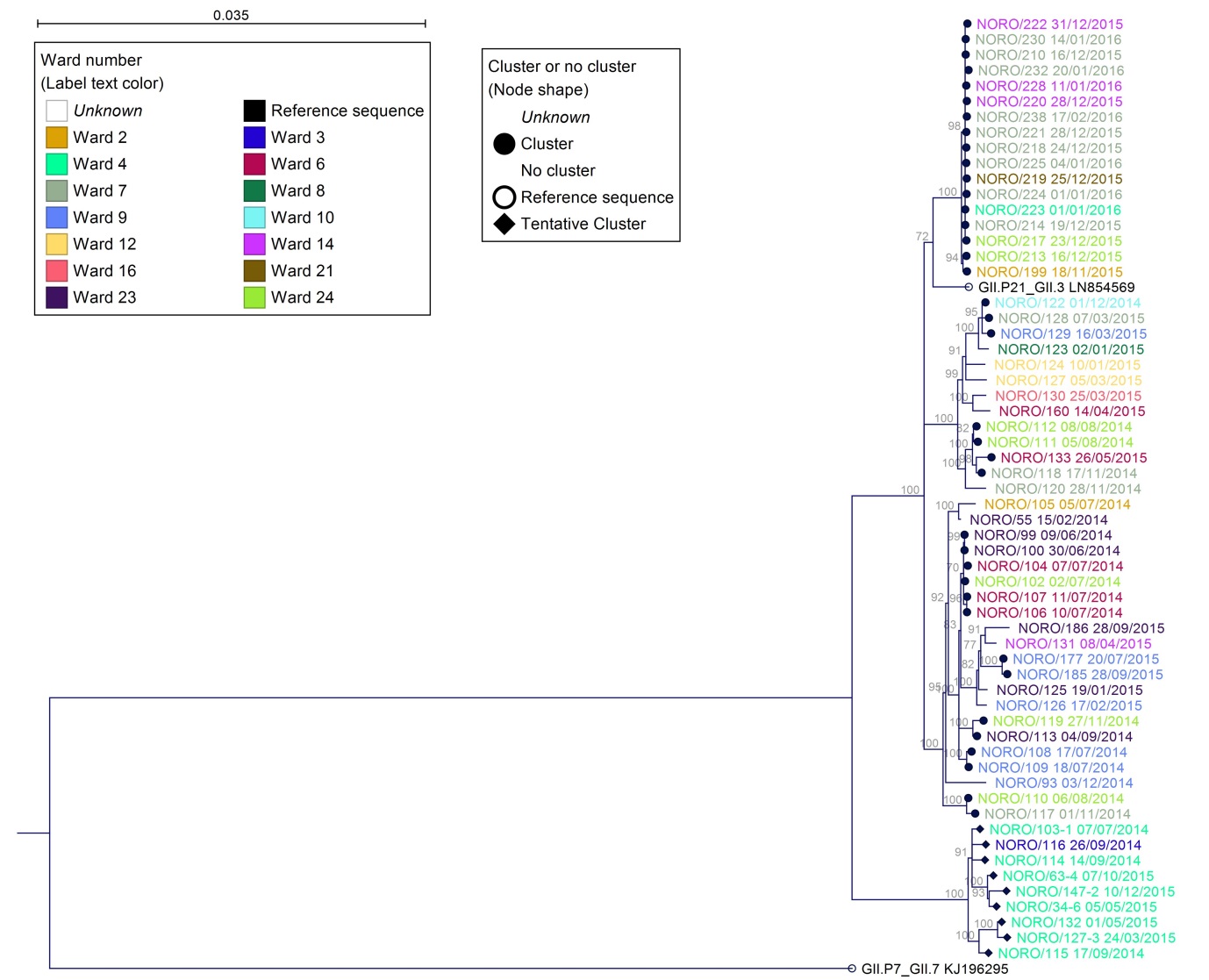


**Cluster 9**

**Cluster 5**

**Cluster 21**

**Cluster 6**

**Cluster 22**

**Cluster 7**

**Cluster 8**

**Cluster 23**

**Cluster 24**

***Tentative Cluster 10***

**(f)**


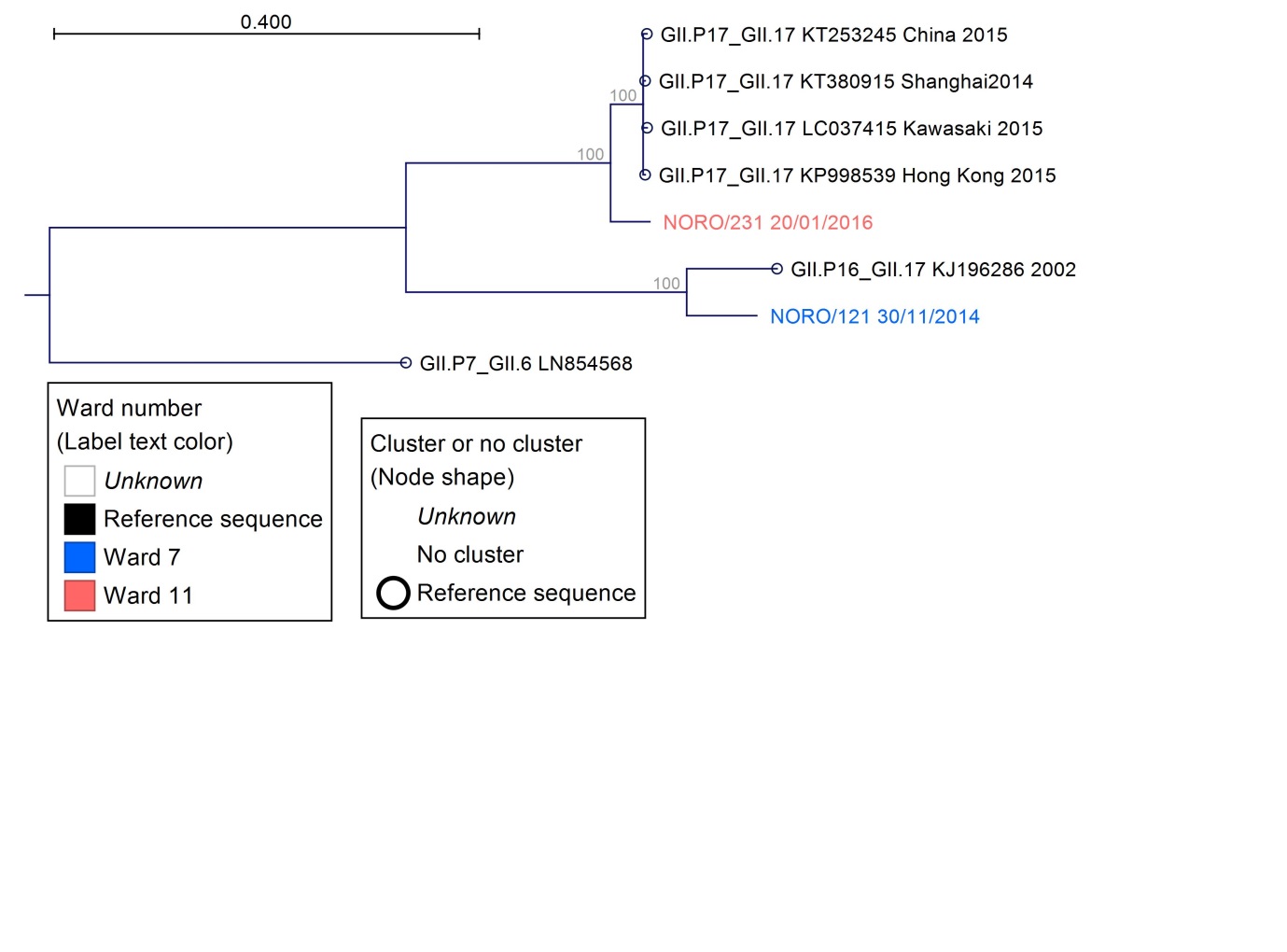


**(g)**

**
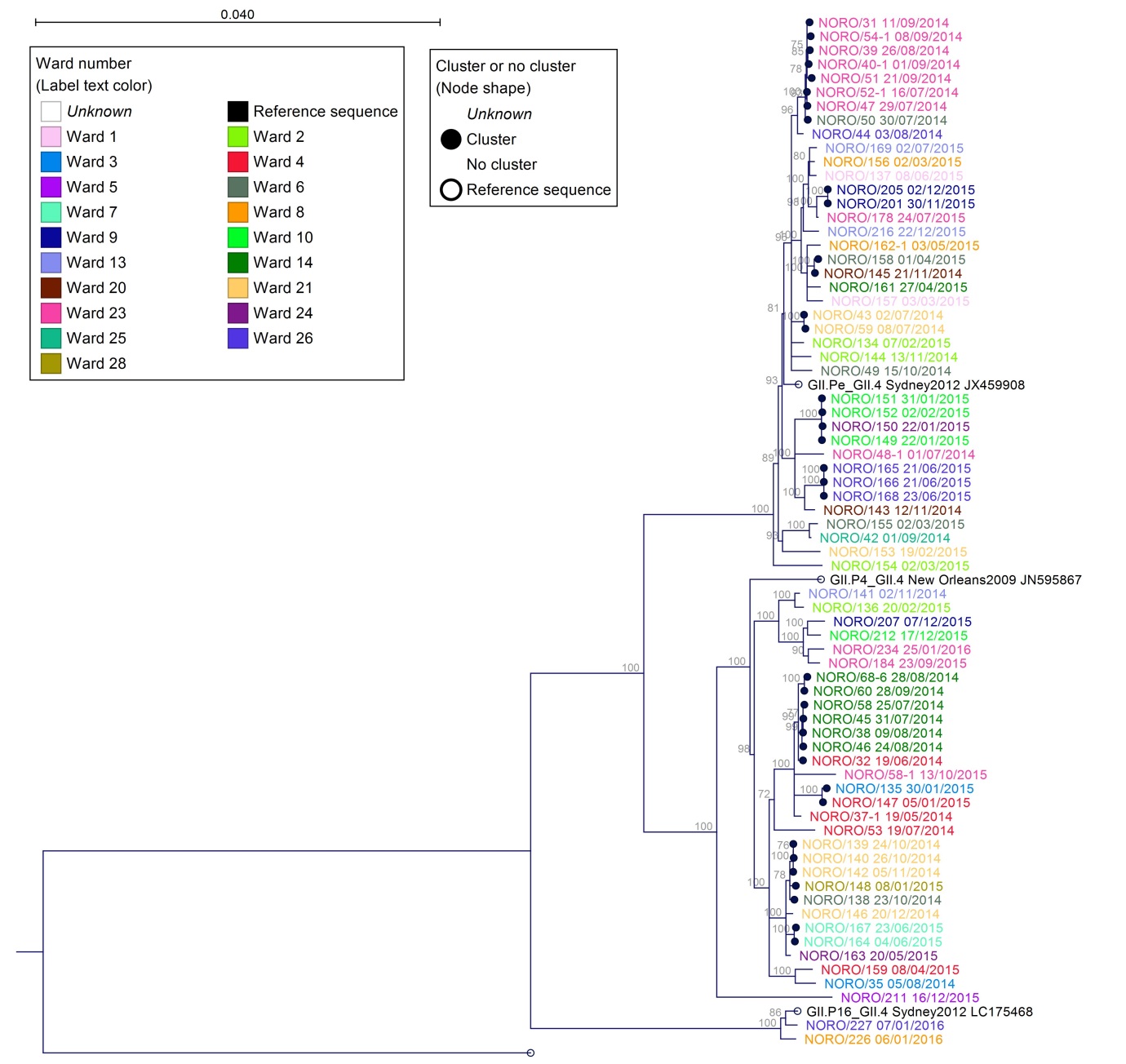
**

**Cluster 11**

**Cluster 12**

**Cluster 13**

**Cluster 14**

**Cluster 15**

**Cluster 18**

**Cluster 19**

**Cluster 16**

**Cluster 17**

**Cluster 20**

**Supplementary Figure 6. Relationship between patients in (a) epidemiologically supported sequence clusters, (b) epidemiologically unsupported sequence clusters and, (c) cluster 10**

**c)**

Numbers in bold indicate patient number, SNPs indicate the number of pairwise single nucleotide polymorphisms between full genomes. The days between specimens are shown for cluster 10 (Figure 6c).

**
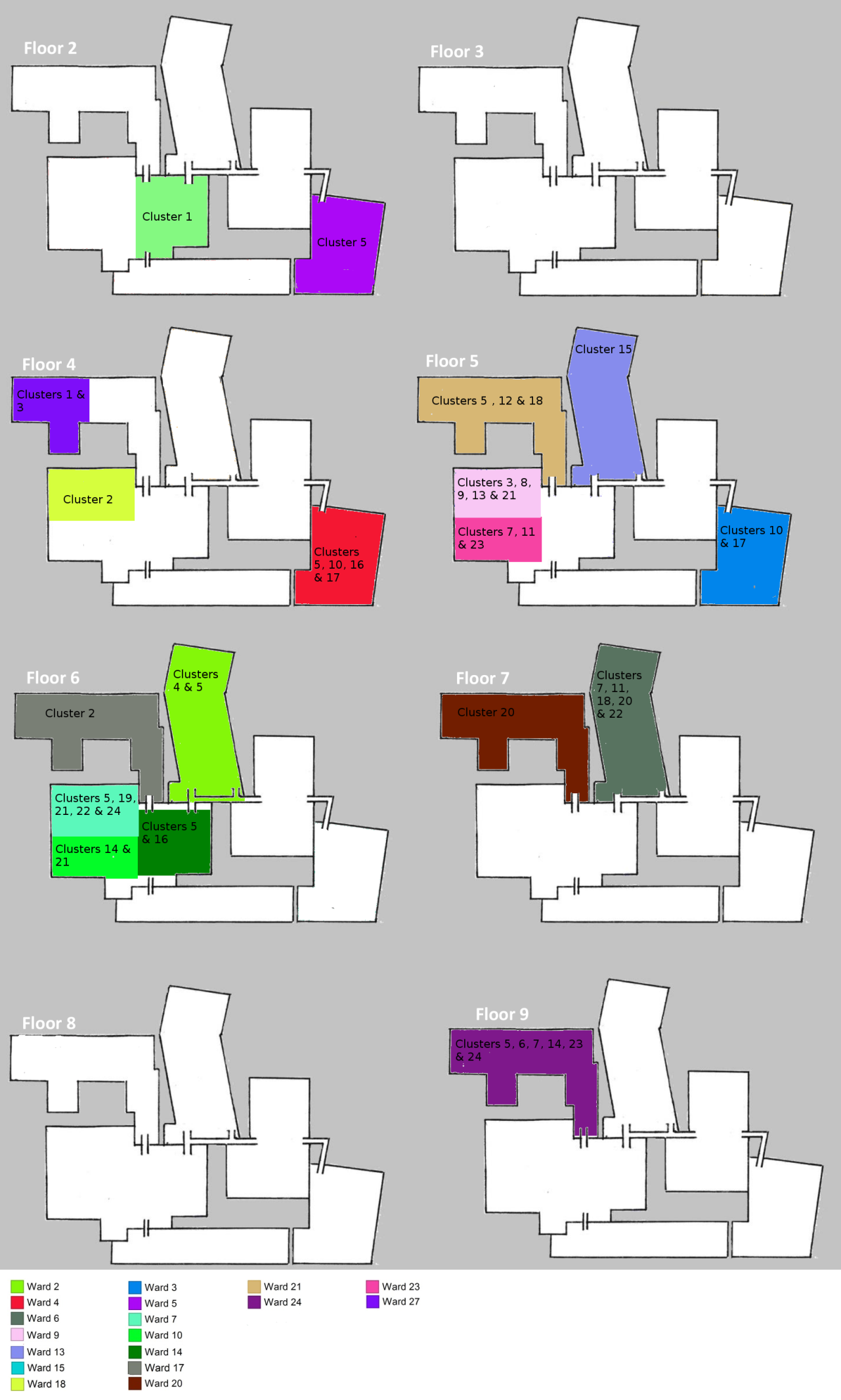
**

**Supplementary Figure 7:** Relationship between clusters and wards in the hospital

**Supplementary Figure 8.** Maximum likelihood phylogeny of full genome sequences from norovirus episodes with a **GII.3** capsid genotype, including all publically available GII.P21_GII.3 sequences. Individual sequences are labelled with a unique patient identifier (NORO/XX) and specimen collection date or GenBank accession number if publically available sequence.

**
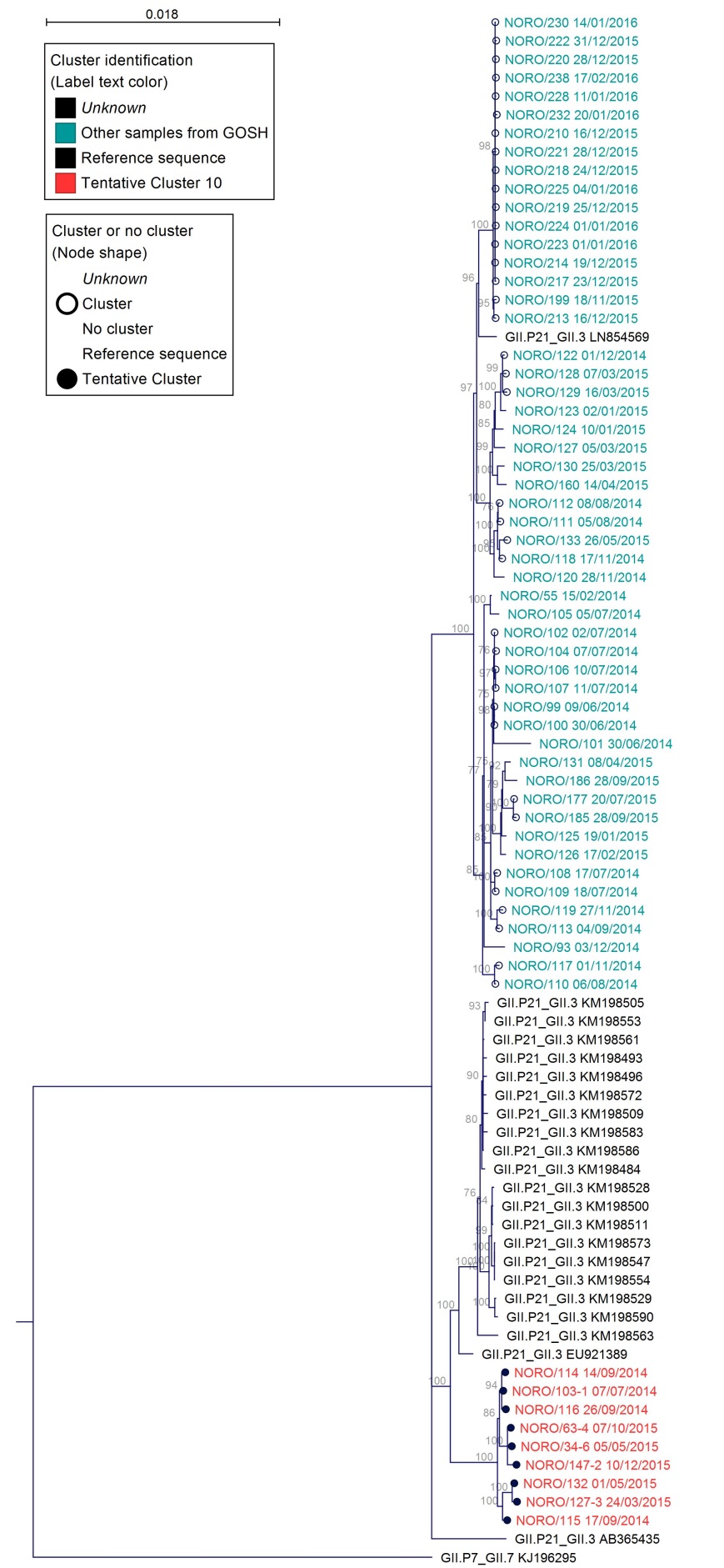
**

**Supplementary Figure 9:** Decision Tree based on combining Norovirus Sequencing and Epidemiological Data
