# Supplementary material for: Norovirus Transmission Dynamics in a Pediatric Hospital Using Full Genome Sequences
Source: Clin Infect Dis. 2018 May 25;68(2):222–8. doi: 10.1093/cid/ciy438 (PMC6321856; doi:10.1093/cid/ciy438)
Supplement: Supplementary Methods [file ciy438_suppl_supplementary_methods.docx]

**Norovirus transmission dynamics in a paediatric hospital using full genome sequences**

Julianne R Brown^1^, Sunando Roy^2^, Divya Shah^1^, Charlotte A Williams^2^, Rachel Williams^2^, Helen Dunn^1^, John Hartley^1^, Kathryn Harris^1^ and Judy Breuer^1,2^

1 Microbiology, Virology and Infection Prevention and Control, Great Ormond Street Hospital NHS Foundation Trust, UK

2 Infection and Immunity, University College London, UK

**SUPPLEMENTARY METHODS**

*Management of patients with norovirus (supplementary figure 1)*

Patients who are symptomatic or who are norovirus positive on stool PCR are isolated in single bedded rooms, with enteric barrier nursing precautions and are sampled weekly until they are PCR negative. All children admitted for haematopoietic stem cell transplant or with congenital immunodeficiencies are isolated regardless of norovirus status. Each isolation room has a positive pressure ventilated lobby leading to a negative pressure room and en-suite private bathroom. The number of staff and visitors entering the rooms is kept to a minimum and all wear disposable gloves and gowns. On a daily basis the Infection, Prevention and Control (IPC) team (i.e. the director of IPC (a clinical microbiologist), three IPC nurses and one IPC Clinical Scientist) are informed by the diagnostic laboratory of all new PCR positive cases of norovirus. At the same time, wards report all new cases of vomiting and/or diarrhoea among patients, staff and visitors. Two or more patient. staff or carer cases occurring on the same ward (see supplementary figure 1) are considered a potential nosocomial transmission unless either or both are within 48 hours of admission. Standard control measures including the instigation of enteric barrier precautions are put in place: any communal areas the child may have visited are closed and cleaned and staff are asked to assess families to find out if they have been unwell; if they have, they are asked to go home and not return until they have been symptom free for 48hrs. More than two putative nosocomial cases leads to the declaration of a suspected IPC outbreak, and an outbreak control meeting. As part of a declared outbreak, wards are to assess all patients, carers and staff for symptoms for the duration of the outbreak; wards are closed or placed on restricted visiting, which means that only essential staff and parents should visit the area; cleaning of patient and staff communal areas takes place including playrooms, bathrooms, nursing stations and food preparation areas; staff are reminded not to eat and drink in patient areas; bed managers and senior teams are informed of the outbreak and asked to cascade to colleagues, as well as informing Public Health England (PHE) as part of regional surveillance; Bank staff are restricted and not allowed to work between affected and non-affected areas.

*Phylogenetic analysis*

Consensus sequences were aligned using the very accurate (slow) progressive alignment tool in CLC Genomics Workbench (v 9.0). Maximum likelihood phylogenies were reconstructed using the general time reversible (GTR) nucleotide substitution model, including rate variation (+G), tree topology estimation (+T) and 200 bootstrap replicates. The GTR+G+T model was determined to be the best model for the data set by the Model Testing tool in CLC Genomics Workbench. Consensus sequence alignments were used to calculate pairwise distances (i.e. the number of consensus sequence single nucleotide polymorphisms, SNPs, between genome sequences).

Maximum likelihood trees were reconstructed using full genome sequences for all 182 sequences together and also separately for each genotype. All nodes with bootstrap support <70 were collapsed. Sequence clusters were identified using the maximum likelihood phylogenies reconstructed for each genotype separately, due to increased resolution of the phylogenies.

P2 domain sequences were derived by aligning all sequences to a GII.Pe_GII.4 Sydney_2012 reference sequence (Genbank accession number JX459908); the alignment was trimmed to nucleotides 5910–6336 of the reference sequence, which corresponds to the P2 domain.

The program PopART v1.7 <http://popart.otago.ac.nz/index.shtml> was used to determine the temporal and spacial relationships between patients in each sequence cluster.

*Pairwise distances between genome sequences*

The pairwise distance between two genomes is defined as the total number of positions across the genome at which the two consensus level sequences differ relative to one another ; this is measured in single nucleotide polymorphisms (SNPs), i.e. the number of consensus-level single nucleotide differences between two genomes.
