# Supplementary figures and images for: Norovirus Transmission Dynamics in a Pediatric Hospital Using Full Genome Sequences
Source: Clin Infect Dis. 2018 May 25;68(2):222–8. doi: 10.1093/cid/ciy438 (PMC6321856; doi:10.1093/cid/ciy438)

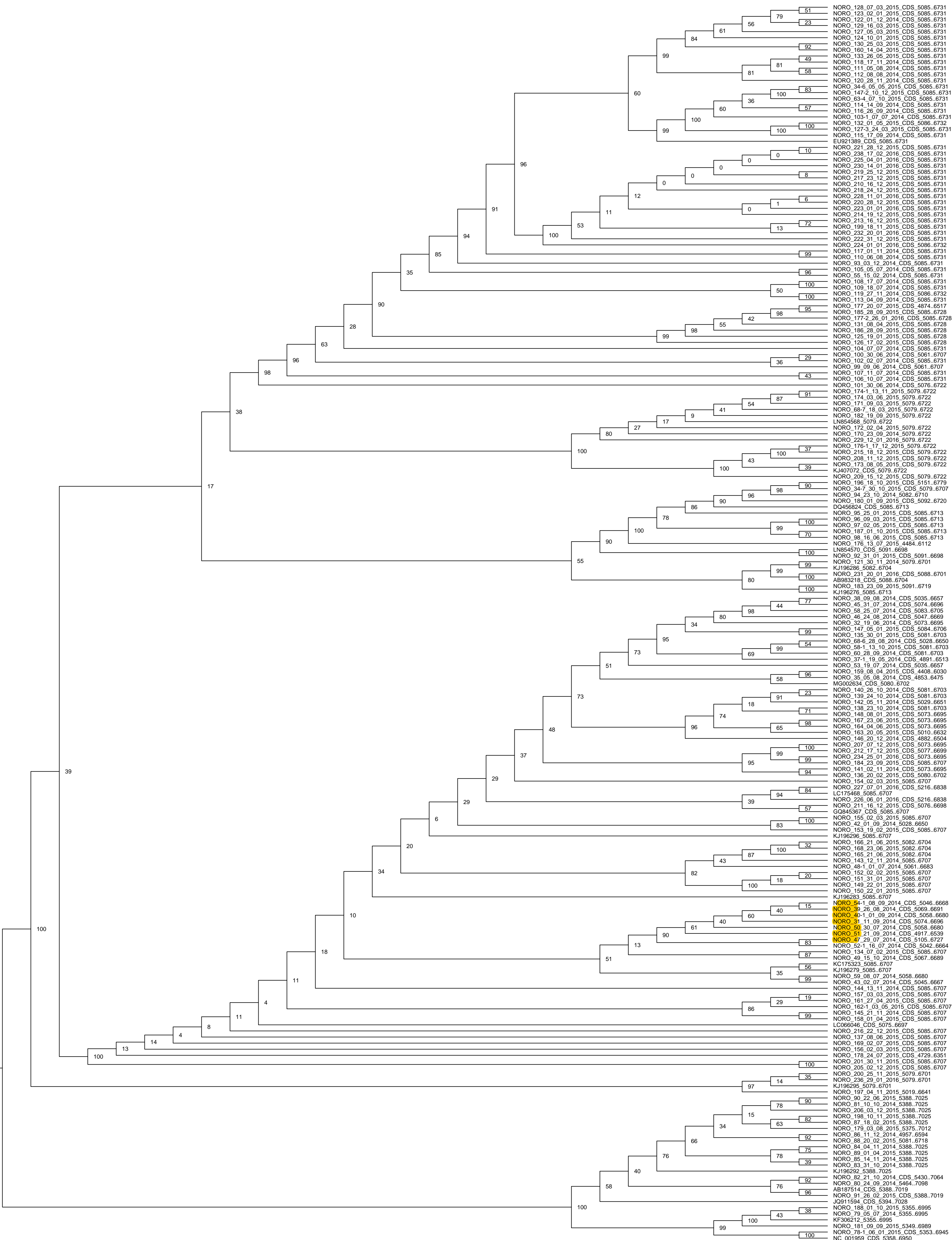

Supplement: Supplementary Figure [file ciy438_suppl_supplementary_figure-tree.pdf]
